# Metallo-beta-lactamase α-helix fragment modified peptide N10 is a multifunctional antimicrobial peptide

**DOI:** 10.1128/spectrum.03040-25

**Published:** 2026-07-17

**Authors:** Hao Lu, Zhaoran Zhang, Fuwei Zhao, Wenjia Lu

**Affiliations:** 1 College of Pharmacy, Heze University 226510, , Heze, China; 2 National Key Laboratory of Agricultural Microbiology, College of Veterinary Medicine, Huazhong Agricultural University 47895, Wuhan, Hubei, China; Universidad Maimonides, Buenos Aires, Argentina

**Keywords:** AMPs, MBLs, cyclic peptide, antibacterial activity

## Abstract

Metallo-beta-lactamases (MBLs) increase the spread of drug resistance among bacteria worldwide and have severely limited the efficacy of β-lactam antibiotics. To develop alternative therapeutics, we used the MBL α-helix fragment as the original peptide, which is the main reason for carbapenem resistance, and modified it into D-amino acid to replace cyclic peptide N10. The antibacterial effect of N10 was detected through the broth microdilution method and time–kill curve and growth curve detection, and the effective minimum inhibitory concentrations (MICs) of N10 to bacteria ranged from 2 to 16 μg/mL. Based on the fluorescence microscopy, hydrogen ion SEM and TEM results, N10 showed extensive membrane damage and structural collapse. In the cell infection model, N10 decreased the levels of ROS and inflammatory factors to near-control levels at 2× MIC. *In vivo*, there was no apparent cytotoxicity or hemolytic activity even at 512 μg/mL. The “0–2–12 h” therapeutic strategy also resulted in noticeable therapeutic effects in a carbapenem-resistant *Escherichia coli*-infected mouse model. Our research revealed a new antimicrobial peptide source and modification strategy, which broadens the antibacterial mode and therapeutic procedure.

**IMPORTANCE:**

In this study, we modified an metallo-beta-lactamase family protein into the novel, safe, environmentally friendly antimicrobial peptide N10, which has antimicrobial, antioxidant, and anti-inflammatory properties both *in vivo* and *in vitro*. We also investigated the preliminary antimicrobial mechanism of the biopeptide. This study broadens the use of drug resistance genes and provides direction for *in vivo* therapeutic strategies.

## INTRODUCTION

Drug resistance has caused substantial economic losses to public health and aquaculture (1), an issue that has been among the greatest concerns of related industries in recent years (2, 3). Alternative antibacterial agents have become a key area of research. The development process of traditional antibiotics is both slow and expensive, and drug resistance mutations represent a significant challenge in this approach. Antimicrobial peptides (AMPs) are being rapidly developed because of their environmental sustainability and cost savings (4). The main mechanisms underlying the bactericidal effects of AMPs can be divided into membrane-targeting, intracellular, antibiofilm, and immunomodulatory mechanisms (5). Currently, artificial intelligence has been used to develop this field. For example, Mousa et al. (6) discovered a group of AMPs containing an average of 35–57 amino acids in microbial derivatives with good antimicrobial effects; diffusion modeling was combined with molecular dynamics to obtain AMPs with antibacterial or antifungal activity (7); and *in vitro* and computerized strategies were employed to modify AMPs to improve the stability of AMPs (8). Even though novel methods of AMP development have efficient screening capabilities, the traditional approach remains meaningful because of its controllability. Metallo-beta-lactamases (MBLs) are a family of proteins with highly conserved motifs that are widely found in gram-negative bacteria (9). This protein family has various activities, such as nuclease activity, anticancer drug degradation, and membrane transport (10, 11). MBL family proteins can hydrolyze cephalosporins, penicillins, and carbapenem antibiotics (12). The main research on MBL proteins involves functional exploration. For example, the MBL fold metallo-hydrolase nftF1 can influence the synthesis of bacterial aromatic polyketones (13), and MBL proteins have been shown to participate in arsenic resistance processes in acidophilic bacteria (14); moreover, it is connected to translation in giant viruses (15). However, the function potential of MBL proteins goes far beyond that. To date, no one has used MBL protein fragments as templates for AMP design (16). MBL proteins are rarely modified into AMPs because (i) the enzyme structure and catalytic sites of MBL are complex, (ii) the enzymes exhibit poor template stability, and (iii) the mechanisms of enzymes and AMPs differ significantly (17). As indicated in our previous researches, we found that bony fish chemokine and T6SS-related protein Tsap can be modified into AMPs (18–20). Broad-spectrum bactericidal activity with low cytotoxicity can be achieved through rational enhancement of positive charge and amphipathic α-helical structures. The antimicrobial efficacy of these derivative peptides against multidrug-resistant bacteria has been systematically validated, and their underlying mechanisms were elucidated, primarily involving membrane disruption and ROS induction. However, whether MBL also has the same potential is unknown.

To further enhance the use of the bacteria’s own weapon as an antimicrobial source, we first modified the MBL helical peptide into an AMP with antibacterial effects, N10, which not only possesses broad-spectrum antimicrobial effects and no cytotoxicity or hemolytic activity but also has the ability to cause drug resistance mutations in bacteria. N10 has been engineered to scavenge bacteria by disrupting the structure of cellular membranes. This, in turn, leads to an increase in intracellular ROS levels and the subsequent leakage of ATP. Its anti-inflammatory ability and *in vivo* antibacterial effect also demonstrated the significant potential of N10 as a novel antibacterial agent. This study further expands the sources of AMPs and provides a theoretical basis for the modification of AMPs and *in vivo* antimicrobial therapy. Our research highlights the scope of AMPs and the connections between AMPs and drug targets. Moreover, this study provides direction for antimicrobial therapy strategies *in vivo*.

## RESULTS

### MBL fragments can be modified into AMPs

In a continuation of our previous study, we focused on modifying the intrinsic defense mechanisms of bacteria into AMPs with antibacterial effects (21). We transformed the helical segments of MBLs into AMPs N1–N10. Among these, N8 and N10 exhibited excellent antibacterial activity both in standard and multidrug-resistant strains (Table 1).

**TABLE 1:** N, N1–N10 antimicrobial activity against bacteria

MIC (μg/mL)									
		Standard strains		MDR strains					
Name	Sequence	*E. coli ATCC 25922*	*S. aureus ATCC 25923*	*E. coli 250321*	*Streptococcus SC19*	*E. coli 42*	*E. coli 200*	*S. aureus 2117*	*S. suis 16072*
N	RRLCPIPF	>128	>128	>128	>128	>128	>128	>128	>128
N1	RRRCPIPR	>128	>128	>128	>128	>128	>128	>128	>128
N2	RRLCPPFF	64	64	>128	>128	>128	>128	>128	>128
N3	RRLCPIPP	>128	>128	>128	>128	>128	>128	>128	>128
N4	KRLCPIPK	32	32	64	32	128	128	64	128
N5	RRLCPCPF	64	>128	>128	>128	>128	>128	>128	>128
N6	RRLWPIPF	>128	>128	>128	>128	>128	>128	>128	>128
N7	RRICPIIF	64	64	>128	64	>128	>128	128	128
N9	RRLCPLPF	32	16	64	32	64	64	64	64
N8	(dR)(dR)L(dC)PIPF	8	8	32	8	64	32	32	32
N10	(dR)(dR)L(dC)PIPY	4	2	8	4	8	8	8	4

**Fig 1:**
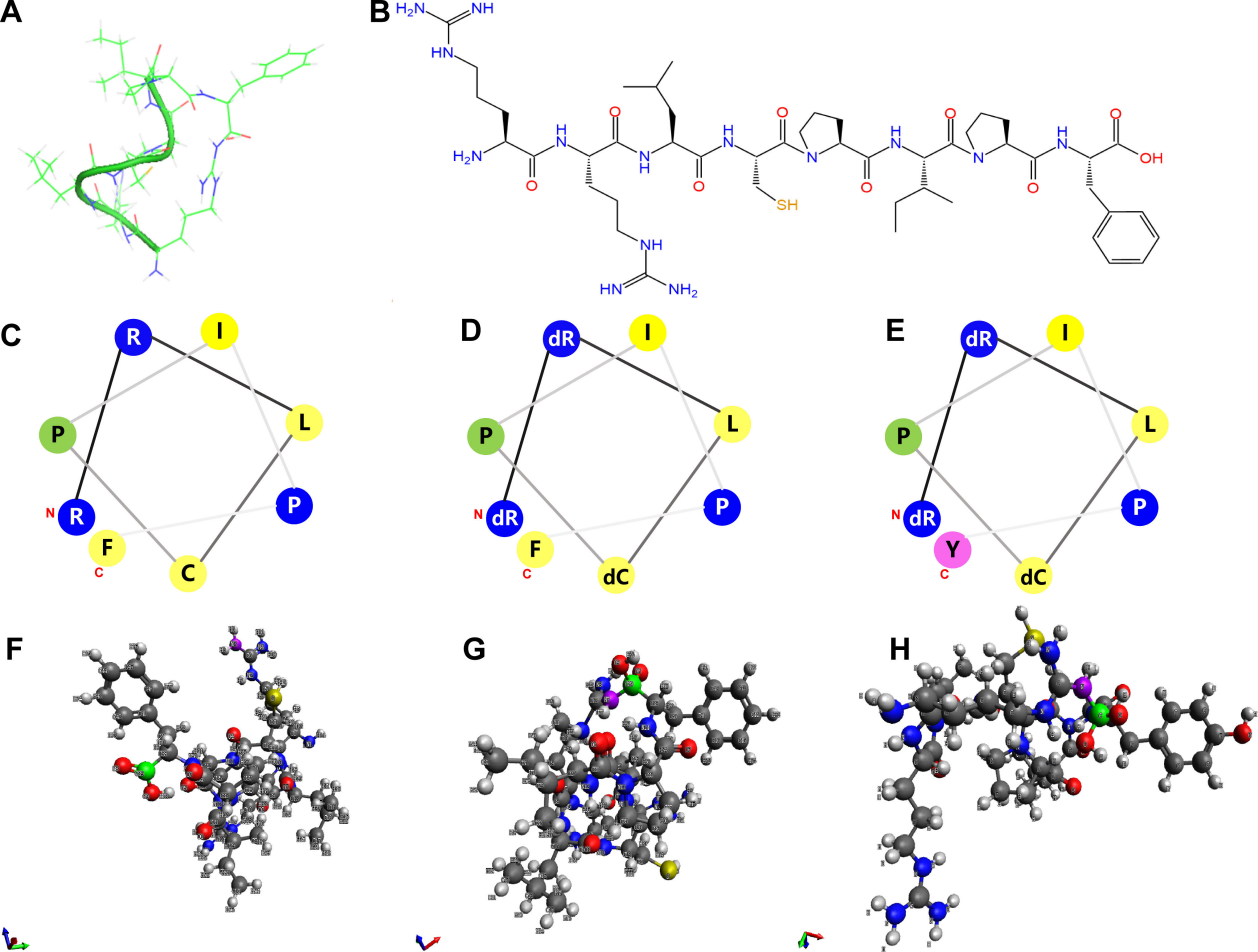
MBL fragements can be modified into AMPs. (**A**) The 3D structure of peptide N. (**B**) Chemical formula of the original peptide. The helix wheels of N peptide (**C**), N8 peptide (**D**), and N10 peptide (**E**). Stick space models of the original peptide (**F**), N8 peptide (**G**), and N10 peptide (**H**). N-atoms are marked in blue, red represents O-atoms, pink represents the C-terminal carboxyl C atom, and the N-terminal amino N atom is in green.

The selected MBL fragment N (RRLCPIPF) contains a typical α-helical segment (Fig. 1A), with the chemical structural formula shown in Fig. 1B. A helix wheel diagram of the peptide N (Fig. 1C) and modified peptides N8 and N10 (Fig. 1D and E) revealed the amino acid linkage of the peptides. Stick diagrams were generated to illustrate the spatial structures of the native linear peptide (Fig. 1F) and cyclic peptides N8 and N10 (Fig. 1G and H), where the N- and C-terminal amides of N8 and N10 form a ring.

These three peptides contain eight amino acids, and the amino acid composition of the peptide is RRLCPIPF, with Arg and Cys at positions N8 and N10 replaced by D-amino acids. The structural basis for the antimicrobial activity of N8 and N10 can be attributed to three synergistic design elements: an electrostatic core formed by the dArg-dArg (dR-dR) motif, a hydrophobic and cyclization-stabilized center contributed by d-Cys, and an aromatic membrane-anchoring residue provided by Tyr. Together, these features endow N10 with enhanced membrane affinity, structural stability, and bactericidal potency.

In accordance with the basic properties listed in Table 2, in an environment with pH = 7.0, the net charge of N10 is 1.9. The grand average of hydropathy (GRAVY) value was calculated as −0.34. These characteristics indicate that N10 might be an excellent antimicrobial peptide.

**TABLE 2:** Basic properties of peptides N, N8, and N10^*a*^

Name	Sequence	Length	Average molecular weight (g/M)	GRAVY	Chemical formula	Net charge
Original peptide	RRLCPIPF	8	1,001.25	0.18	C46H76N14O9S	1.9
N8	(dR)(dR)L(dC)PIPF	8	1,001.25	0.18	C46H76N14O9S	1.9
N10	(dR)(dR)L(dC)PIPY	8	1,280	−0.34	C46H76N14O10S	1.9

^*a*^ GRAVY: grand average of hydropathy.

### N10 has neither cytotoxicity nor the ability to induce drug-resistant mutations

**Fig 2:**
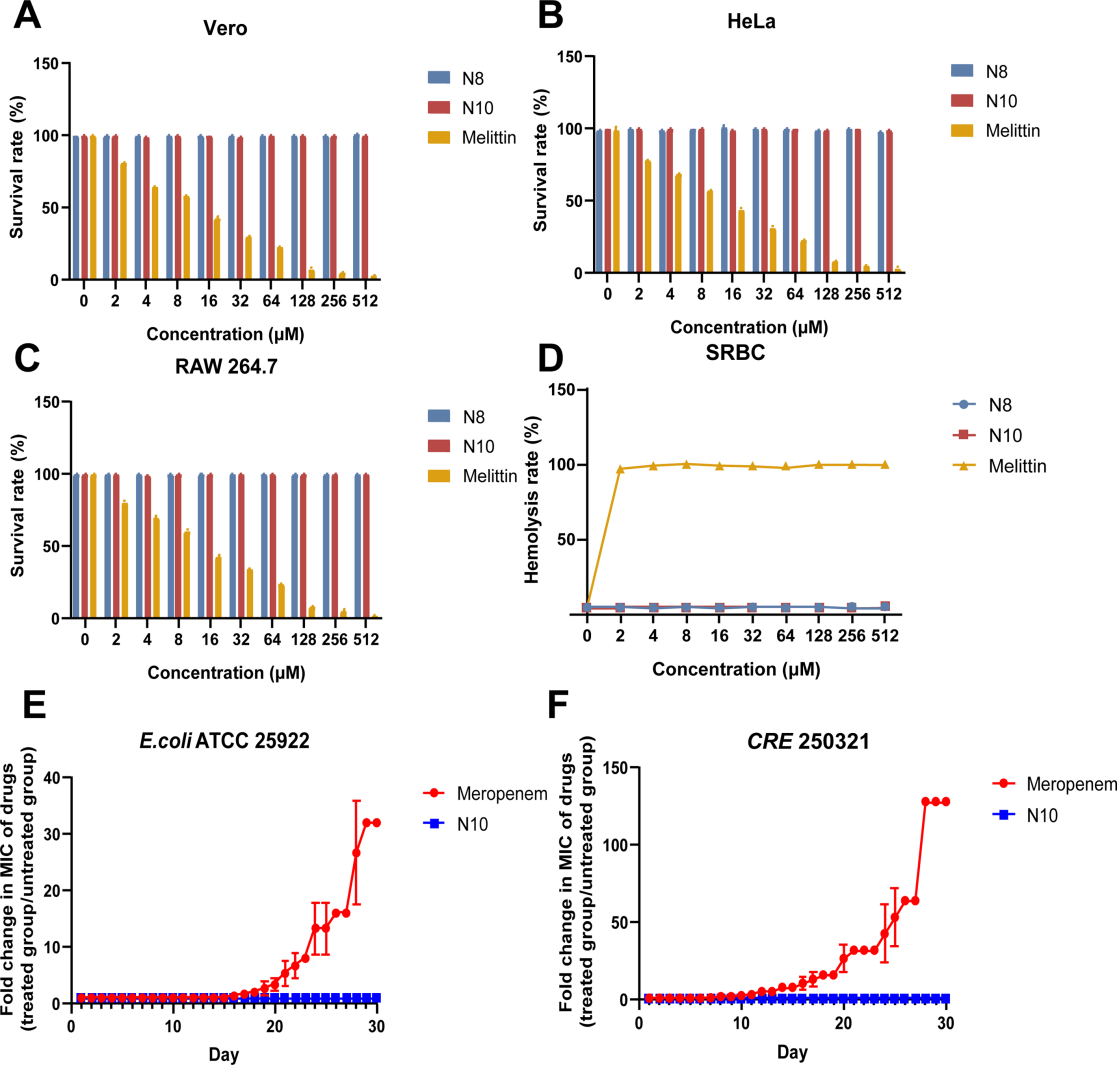
N10 neither has cytotoxicity nor the ability to induce drug-resistant mutations. (**A–C**) Cytotoxicity to Vero, HeLa, and RAW264.7 cells after treatment with 0, 2, 4, 8, 16, 32, 64, 128, 256, and 512 μg/mL of the peptides N8 and N10 and the control drug melittin. (**D**) Hemolytic activities of the peptides N8 and N10. Fresh sheep erythrocytes were mixed with 0, 2, 4, 8, 16, 32, 64, 128, 256, or 512 μg/mL N8 or N10 peptide at 37°C. The same concentrations of melittin were used as positive controls. (**E**) Drug resistance testing of meropenem and N10 in *E. coli ATCC 25922*. (**F**) Drug resistance testing of meropenem and N10 in *CREC 250321*.

To verify the safety of N10, human-derived Vero and HeLa cells, as well as mouse-derived RAW 264.7 cells, were used to determine whether the peptides are cytotoxic. After treatment with 0–512 μg/mL peptides, the system had no significant effect on the cytotoxicity of either N8 or N10, and the survival rates of the three cell lines exceeded 97.3%. In contrast, at a concentration of 2 μg/mL of melittin, the survival rates of VerO, HeLa, and RAW264.7 cells were 80.83%, 77.82%, and 80.27%, respectively. Moreover, in the presence of 32 μg/mL melittin, the survival rates of all three cell lines were less than 50% (Fig. 2A through C). Melittin also had significant hemolytic activity at 2 μg/mL (hemolysis rate = 96.9%); however, N8 (hemolysis rate = 4.66%) and N10 (hemolysis rate = 4.84%) demonstrated no hemolytic toxicity even at 512 μg/mL (Fig. 2D).

Overuse of antibiotics can lead to the development of drug resistance. To determine whether N10 had similar side effects, we determined the bacterial resistance ability by the successive passages method. After 30 days of serial MIC detection, we found that the MIC of meropenem increased in a stepwise manner as time increased, including a 32-fold increase in *E. coli* ATCC 25922 (Fig. 2E) and a 128-fold increase in *CREC* 250321 (Fig. 2F). However, N10 did not significantly change in either condition (fold change = 1).

### N10 is a stable antimicrobial peptide with significant antimicrobial effects

**Fig 3:**
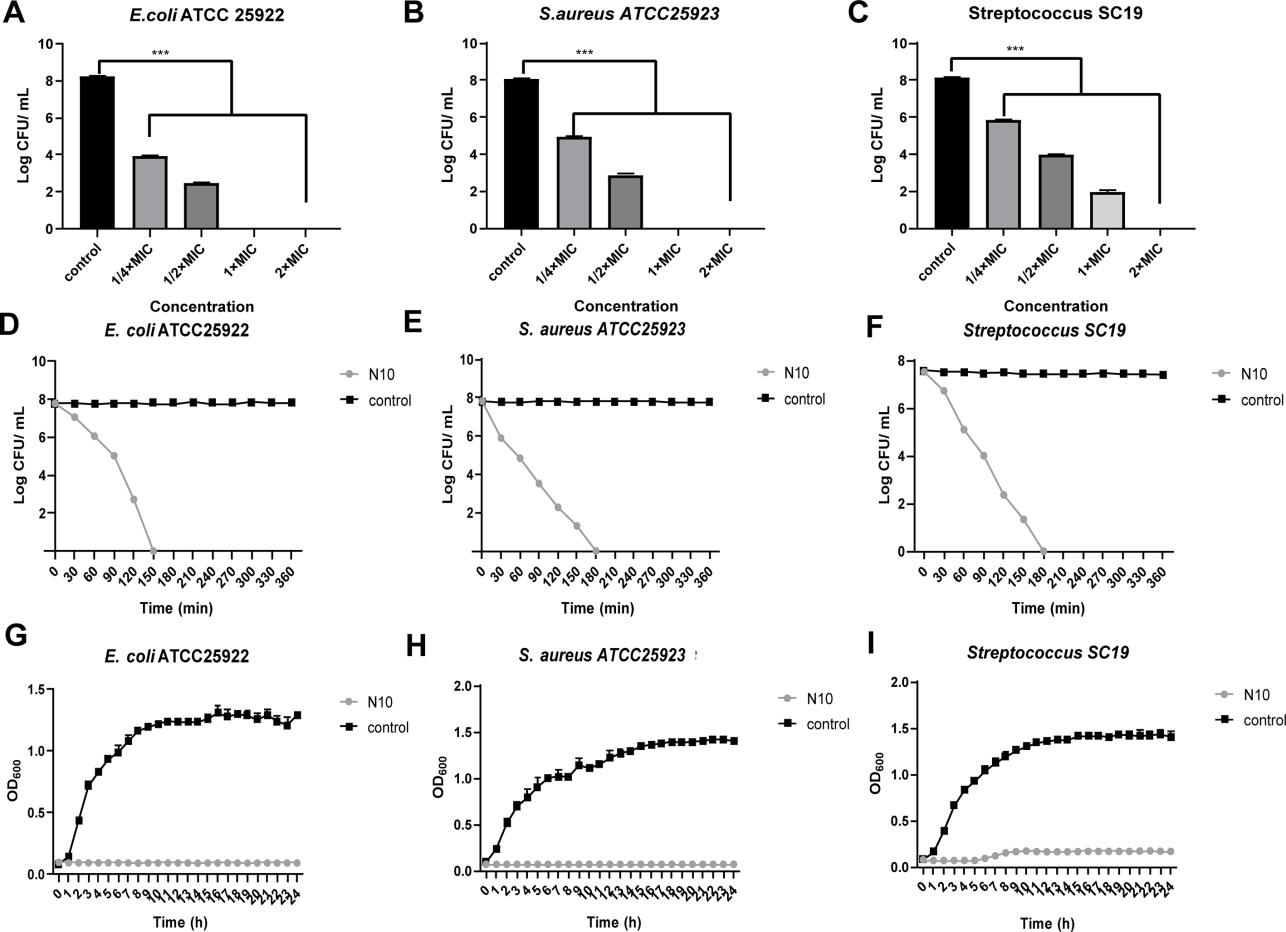
N10 is a stable antimicrobial peptide with significant antimicrobial effects. (**A–C**) Bactericidal activity of N10 against *E. coli* ATCC 25922, *S. aureus* ATCC 25923, and *Streptococcus* SC19. The bacteria were cultured with peptide N10 at 1/4× MIC, 1/2× MIC, 1× MIC, or 2× MIC, and the untreated group was used as a control. The CFUs of the surviving bacteria were counted. (**D–F**) Time‒kill curves of N10 in *E. coli*, *S. aureus,* and *Streptococcus*. The bacteria were mixed with 1× MIC of N10 and cultured at 37°C, and the CFUs were counted every hour for a series of 12 h. The untreated group was used as a control. (**G–I**) Growth curves of *E. coli* ATCC 25922, *S. aureus* ATCC 25923, and *Streptococcus* SC19 strains treated with N10. The OD_600_ of the bacteria treated with 1/2× MIC N10 was continuously measured for 12 h. Student's *t*-test was applied for statistical analysis. Data are presented as mean ± standard error of the mean, significance levels were defined as *P* < 0.001 (***).

To demonstrate the broad-spectrum antimicrobial activity of N10, different concentrations of N10 were used to evaluate the MICs of various strains. The bactericidal abilities of N10 to *E. coli*, *S. aureus*, and *Streptococcus* spp. decreased after 1 h of treatment with 1/4× the MIC of N10. Both *E. coli* and *S. aureus* were completely eradicated by N10 at 1× MIC (4 μg/mL and 8 μg/mL, respectively), whereas *Streptococcus SC19* required 2× MIC (8 μg/mL) to achieve the same bactericidal effect (Fig. 3A through C). The time‒kill curve also revealed that all bacteria were eliminated with 1× MIC of N10 within 3 h (Fig. 3D through F). The growth curve also revealed that these three strains showed marked growth inhibition in the presence of 4 μg/mL N10 (Fig. 3G through I).

Furthermore, N10 retained highly antimicrobial properties in different environments, such as at different osmolarities (water and 2% NaCl), at different pH values (pH = 9 and pH = 5), at 100°C and in the presence of SDS. The specific data are shown in Table 3.

**TABLE 3:** Determination of N10 stability under different conditions

MIC (μg/mL)							
Factor	Control	water	pH = 9	pH = 5	2% NaCl	1% SDS	100°C
*S. aureus* ATCC 25922	2	2	2	2	2	2	2
*Salmonella* 241107	4	4	4	4	4	4	4
*E. coli 250312*	8	8	8	8	8	8	8
*Klebsiella pneumoniae* WH11	8	8	8	8	8	8	8
*Candida albicans* 230001	16	16	16	16	16	16	16

### N10 damage to bacteria occurs mainly through membrane disruption mechanisms

**Fig 4:**
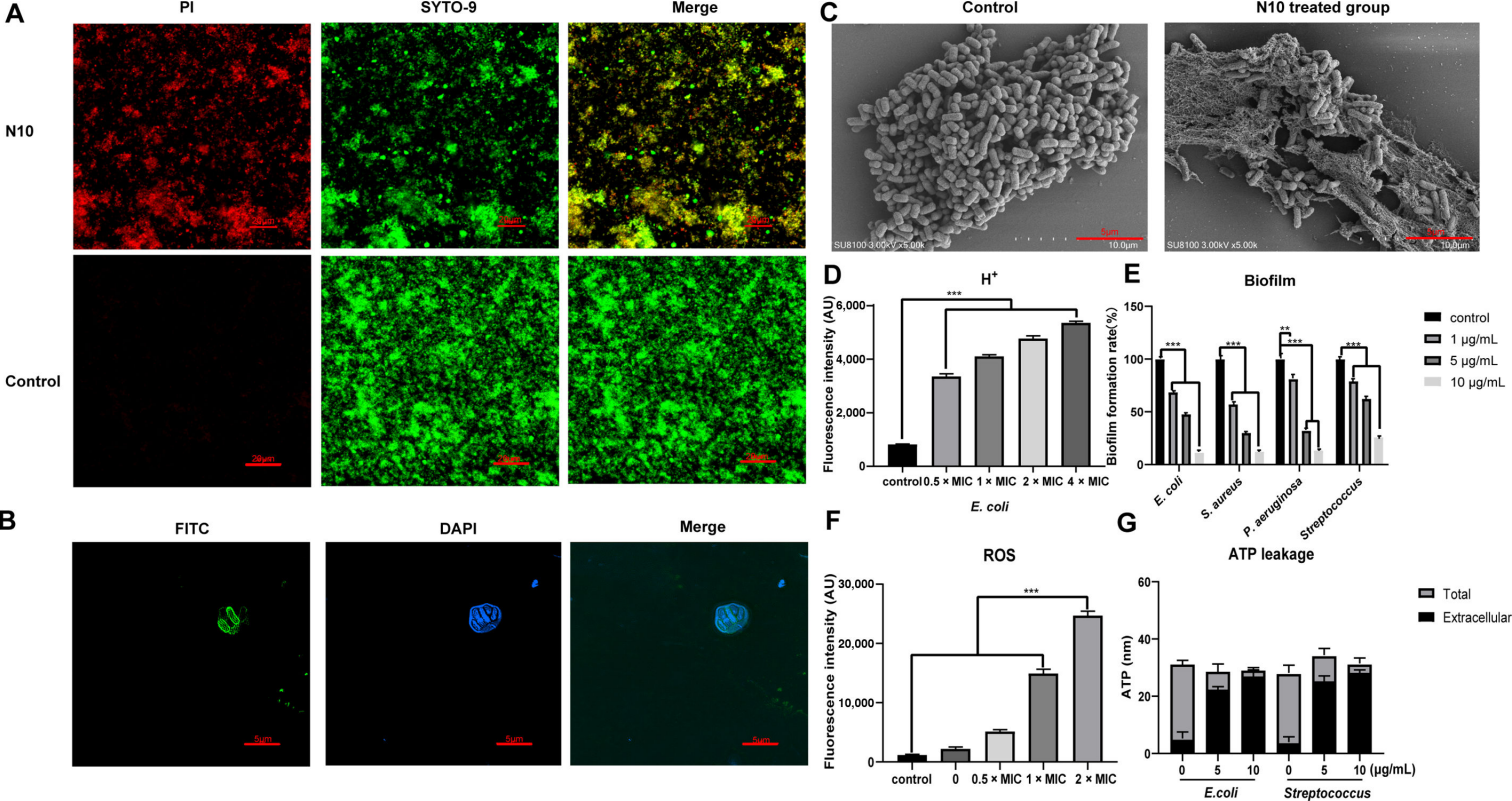
N10 damaged bacteria mainly through membrane breakage mechanisms. (**A**) Biofilm integrity measurement of *E. coli* cultured with peptide N10. The red fluorescence dye PI was used to stain the membranes of dead strains, and SYTO-9 was used to stain live/dead strains in the green fluorescence channel. Scale bars –20 μm. (**B**) Colocalization of FITC-labeled N10 and *E. coli* ATCC 25922. The bacteria were cultured with 0.5× MIC of FITC-N10 for 30 min, and the whole bacteria were stained with DAPI. Scale bars –5 μm. (**C**) *E. coli* morphology before and after N10 treatment via SEM. Scale bars –5 μm. (**D**) Hydrogen ion content determination. The adjusted bacteria were cultured with 0.5×, 1×, 2×, and 4× MIC of N10. An H^+^ probe was then used to detect the H^+^ content. (**E**) Crystal violet staining of bacterial biofilms. Biofilm formation of *E. coli*, *S. aureus*, *P. aeruginosa*, and *Streptococcus* strains cultured with 1, 5, and 10 μg/mL peptide N10. (**F**) Determination of the ROS content after infection with *E. coli* ATCC 25922 and treatment with different concentrations of N10 treatment. Untreated *E. coli* ATCC 25922 was used as control. (**G**) ATP leakage of peptide N10 treated with *E. coli* ATCC 25922 or *S. aureus* ATCC 25923. Student’s *t*-test was applied for statistical analysis. Data are presented as mean ± standard error of the mean, significance levels were defined as *P* < 0.01 (**) and *P* < 0.001 (***).

As demonstrated in previous studies, the key mechanism by which antimicrobial peptides scavenge bacteria involves rupture of the bacterial membrane and leakage of ATP. Furthermore, live cells are stained with SYTO-9 and fluoresce green, whereas dead cells are stained with propylidene iodide (PI) and fluoresce red due to N10-ruptured cell membranes. In the N10-treated group, the PI channel exhibited markedly increased red fluorescence compared to that in the control group, indicating a greater proportion of membrane-compromised bacteria. In contrast, the SYTO-9 channel showed significantly reduced green fluorescence relative to that of the control, suggesting a loss of membrane integrity and decreased viability. The merged images display the overall distribution of bacterial cells and allow visualization of the relative contributions of live and dead populations. The addition of N10 improved *E. coli* and *S. aureus* biofilm removal, as shown in Fig. 4A and Fig. S1A available at https://data.mendeley.com/datasets/9r4rcv6skv/1.

To further explore the mechanism underlying the bactericidal ability of N10, we used FITC-labeled N10 to colocalize the bacteria and visualized N10 via confocal microscopy, in which the green fluorescence signal reflects the positions of bacterial cells at the locations where N10 interacts with them at that time. The results revealed that N10 first localized to the bacterial membrane structure; at that time, N10 crossed the cell membrane into the intracellular space (Fig. 4B; Fig. S1B available at https://data.mendeley.com/datasets/9r4rcv6skv/1). In the N10-treated group, TEM images revealed that the *E. coli* and *S. aureus* cells displayed extensive aggregation, with markedly indistinct or diffuse cell boundaries. These structural alterations suggest severe disruption of the outer membrane and loss of normal cell envelope morphology. N10 caused crumpling and rupture of the bacterial membranes of *S. aureus* and *E. coli* (Fig. 4C; Fig. S1C available at https://data.mendeley.com/datasets/9r4rcv6skv/1).

Proton motive force (PMF) enhances ATP synthesis through a transmembrane proton gradient, and AMPs perforate the membrane, thus increasing H^+^ leakage. We used the H^+^ detection probe BCECF-AM to measure the H^+^ content of *E. coli* in the presence of different MICs of N10. As shown in Fig. 4D, in *E. coli* ATCC 25922, a significant increase in the intracellular H^+^ concentration was observed upon treatment with N10 at 0.5× MIC, and this increase became more pronounced with increasing N10 concentration. These findings indicate that N10 disrupts membrane permeability and that the resulting proton influx occurs in a concentration-dependent manner. Similarly, crystal violet staining revealed that treatment with N10 at 1 μg/mL markedly reduced the staining intensities of *E. coli*, *S. aureus*, *P. aeruginosa*, and *Streptococcus* spp., indicating a loss of cell membrane integrity. This effect became progressively more pronounced with increasing N10 concentration, demonstrating a clear concentration-dependent response (Fig. 4E).

The membrane breakage mechanism is among the important bactericidal mechanisms of AMPs. To determine the function of N10 in this process, N-phenyl-1-naphthylamine (NPN) was used as a hydrophobic probe to investigate outer membrane permeability (22). As the dose increased, the fluorescence intensity gradually increased (Fig. S1D available at https://data.mendeley.com/datasets/9r4rcv6skv/1). As previously mentioned, PI combines with intracellular DNA when the membrane is broken. Similar to the results for NPN, the fluorescence intensity clearly increased when 2 μg/mL N10 was added and gradually increased as the concentration of N10 increased (Fig. S1E available at https://data.mendeley.com/datasets/9r4rcv6skv/1). DiSC3(5), a membrane depolarization indicator, was also used to study membrane disruption (23). The results exhibited a dose-dependent response (Fig. S1F available at https://data.mendeley.com/datasets/9r4rcv6skv/1). SYTOX Green nucleic acid dye does not penetrate unbroken cell membranes but can permeate damaged membranes, serving as an indicator of dead cells within a population (24). The fluorescence intensity increased 10.46-fold after stimulation with 2 μg/mL N10, and under 4, 8, and 16 μg/mL N10 stimulation, the values increased sequentially to 15.12, 19.62, and 31.52 times the original levels, respectively (Fig. S1G available at https://data.mendeley.com/datasets/9r4rcv6skv/1).

*E. coli* ATCC 25922 was used to observe the ability of the AMP N10 to increase the ROS content of N10 (25). After 1 h of N10 treatment, the ROS levels sharply increased. Only the addition of 0.5× MIC of N10 increased the level of ROS by 1.92-fold. As the concentration of N10 increased, the intracellular ROS levels progressively increased, and compared with the control treatment, treatment with N10 at 2× MIC increased the level of ROS to nearly 20-fold. These results indicate that N10 promotes cell death by increasing ROS levels (Fig. 4F).

In accordance with the basic properties of N10, we detected ATP leakage in *E. coli* ATCC 255922 and *S. aureus* ATCC 25923, and the results revealed that 5 μg/mL N10 caused more than 50% ATP leakage in both *E. coli* ATCC 255922 and *S. aureus* ATCC 25923 (Fig. 4G; Fig. S1C available at https://data.mendeley.com/datasets/9r4rcv6skv/1).

### N10 has excellent anti-inflammatory effects

**Fig 5:**
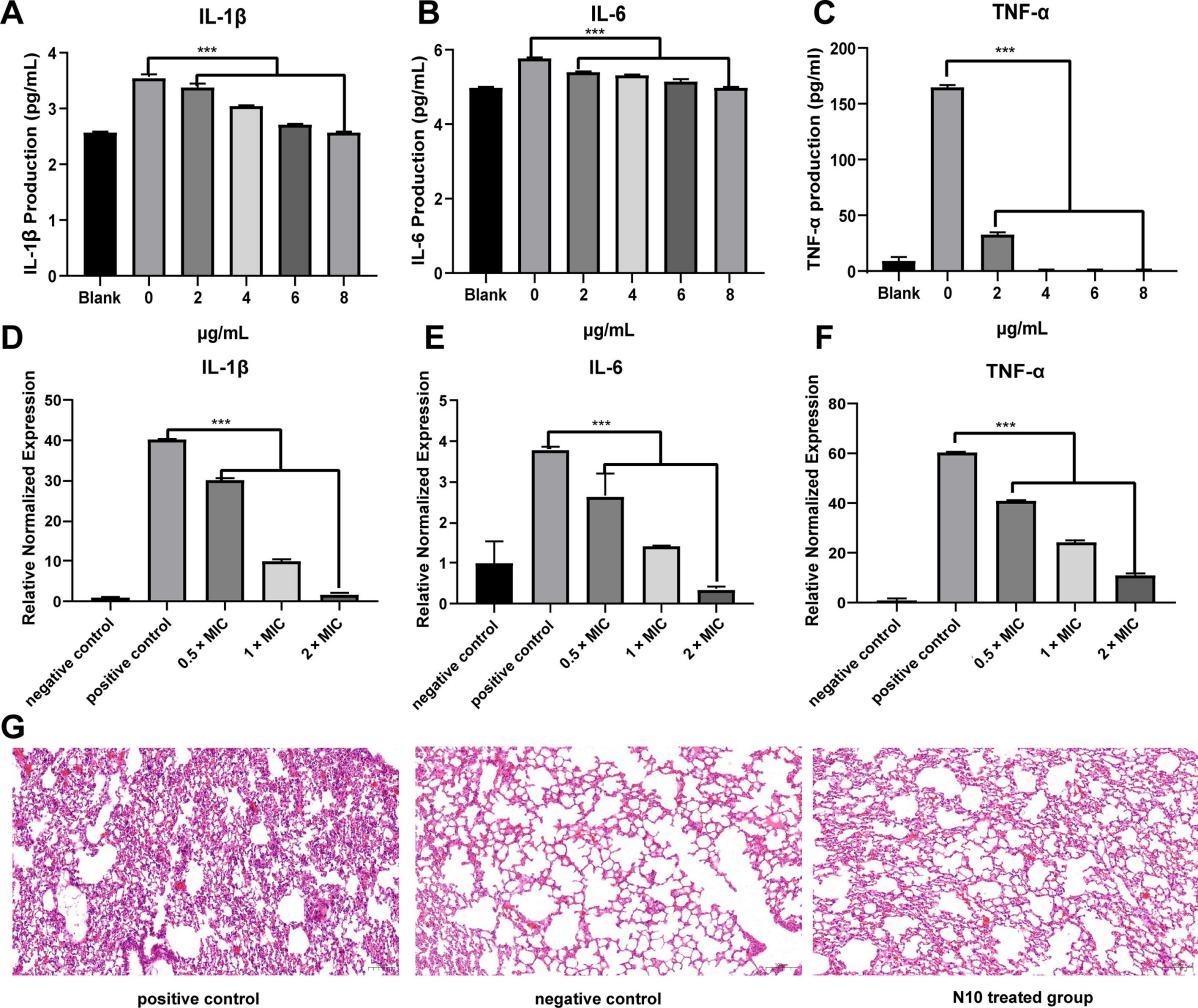
N10 has excellent anti-inflammatory effects. The level content of IL-1β (**A**), IL-6 (**B**), and TNF-α (**C**) in *E. coli*-infected HeLa cells. Relative gene expression levels of IL-1β (**D**), IL-6 (**E**), and TNF-α (**F**) in *E. coli*-infected HeLa cells. (**G**) *E. coli* pneumonia infection model and therapeutic sections. The phosphate-buffered saline (PBS) injection group was used as a negative control, and the infection untreated group was used as a positive control. Scale bars: 100 μm. Student’s *t*-test was applied for statistical analysis. Data are presented as mean ± standard error of the mean, significance levels were defined as *P* < 0.001 (***).

Anti-inflammatory effects are among the significant properties of some AMPs. *CREC* 250321-infected HeLa cells presented significant increases in their IL-1β, IL-6, and TNF-α contents; however, treatment with N10 at 2 μg/mL markedly reduced the levels of these inflammatory cytokines, and at 1× MIC, the cytokine levels decreased to values approaching those of the control group (Fig. 5A and C). This trend was even more pronounced in terms of the relative expression levels of these three cytokines analysis (Fig. 5D through F).

To assess the *in vivo* anti-inflammatory effect of N10, we constructed a mouse intrapulmonary infection model using *CREC* 250321. In the bacterial infection group, diffuse lung tissue was observed within the lungs, with severe destruction of the alveolus pulmonis. Histological sections revealed large amounts of neutrophil and macrophage infiltration, as well as markedly dilated and congested blood vessels. These phenomena were significantly reduced in the N10 treatment group (Fig. 5G).

### Different therapeutic procedures contribute to the role of N10 in the treatment of *E. coli* infections in mice

**Fig 6:**
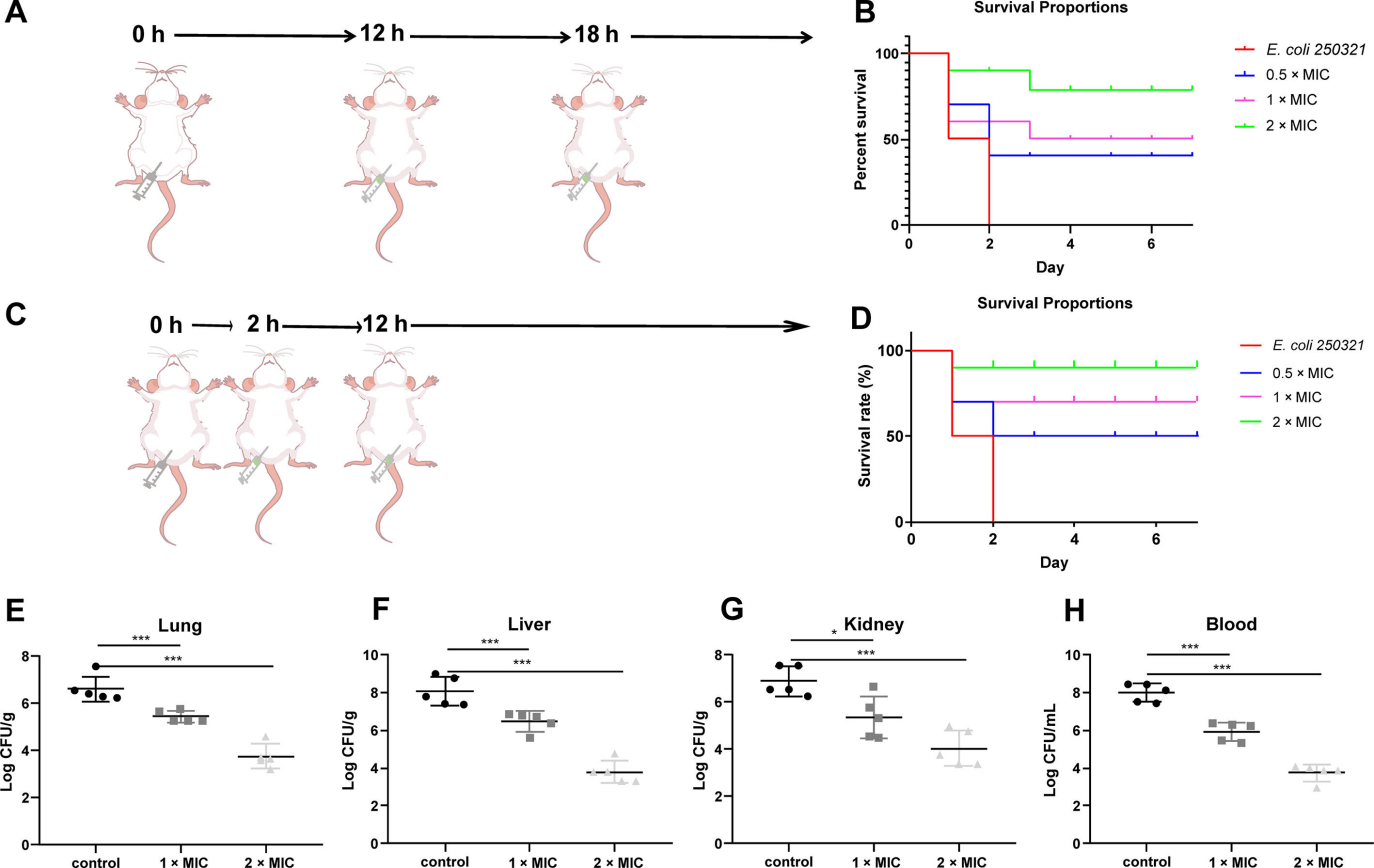
Different therapeutic procedures contribute to the role of N10 in the treatment of *E. coli* infections in mice. (**A**) *E. coli* 250321 mouse intraperitoneal infection model treated at 12 and 18 h post-infection. (**B**) Survival curve of N10 treatment in Model 1. (**C**) *E. coli* 250321 mouse intraperitoneal infection model treated at 2 and 12 h post-infection. (**D**) Survival curve of N10 treatment in Model 2. Bacterial loads in lung (**E**), liver (**F**), kidney (**G**), and blood (**H**). Statistical comparisons were performed using one-way ANOVA followed by Tukey’s multiple comparison test. Differences were considered significant at *P* < 0.05 (*), *P* < 0.01 (**), and *P* < 0.001 (***).

Different therapeutic procedures and models could affect the therapeutic efficacy of the drug; therefore, we used “0–12–18 h” and “0–2–12 h” strategies as therapeutic models (Fig. 6A). The survival rates of mice model treated with N10 at 0.5×, 1×, and 2× MIC were 40%, 50% and 80%, respectively (Fig. 6B). The survival rates of the mice in Model 2 treated with N10 (Fig. 6C) were 50%, 70% and 90%, respectively (Fig. 6D).

In addition, the bacterial tissue loads of the mice indicated that 1× and 2× MIC N10 treatments significantly reduced the bacterial loads in the lung (Fig. 6E), liver (Fig. 6F), kidney (Fig. 6G), and blood (Fig. 6H). These results indicated that N10 has a good therapeutic effect *in vivo*.

## DISCUSSION

Bacterial drug resistance is among the remaining challenges of the 21st century. Currently, overcoming bacterial drug resistance is an essential issue in our daily life. To address this critical issue, we designed the antimicrobial peptide N10 using the α-fragment of the MBL family protein NDM-1 as a template, endowing it with potent antibacterial activity, nontoxicity, and anti-inflammatory effects.

AMPs, such as LL-37, magainin, cecropin, nisin, and As-CATH4 (26), are typically found in mammals, amphibians, insects, microorganisms, and oceans. Research on bacterial drug targets such as carbapenemases (27–29), which can be divided into A, B, C, and D types (30), has received tremendous attention and is essential for research on drug resistance mechanisms. In particular, representative MBL members, including IMP (Imipenemase), VIM (Verona integron-encoded MBL), and NDM (New Delhi MBL), are spreading rapidly around the world (31). Promoting a “drug-resistant element suicide” strategy might become a novel, highly efficient and energy-saving antimicrobial approach. The first step is to modify the peptide into efficient AMPs. Compared with traditional AMPs, N10 modified through NDM-1 represented a novel approach.

Recently, the ways to identify AMPs have diversified and include deep learning combined with quantitative structure (32), multi-CGAN (33), HMAMP (34), and many others. These methods increase the application scope of AMPs; however traditional methods still retain value because of their purposefulness, precision, and safety. According to previous studies, the addition of D-amino acids to peptides might increase their stability and reduce their cytotoxicity (35). The cyclization of AMPs can increase their antimicrobial capacity and their presence on the cell membrane (36). Using these two approaches, we acquired AMP N10, which has improved antibacterial properties.

Compared with melittin, magainin (37), and LL-37 (38), the modified peptide N10 exhibited superior comprehensive antimicrobial performance. Despite their antibacterial ability, cytotoxicity is the main drawback for some AMPs. In our research, we found that N10 has no cytotoxicity or hemolytic activity, even at high concentrations, which is one of the main limitations to the effects of AMPs (39). Importantly, N10 demonstrated antibacterial potency comparable to or stronger than those of several well-studied AMPs, such as LL-37, pexiganan, DP7, and melittin (40), whose MICs typically range from 4 to 32 μg/mL in gram-negative bacteria. This comparative advantage highlights the improved therapeutic potential of N10 compared with those of classical AMPs. Furthermore, N10 retained strong stability under extreme conditions (temperature, pH, and ionic strength), supporting its applicability in complex physiological or environmental settings. According to our infection-treatment Model 2, the survival rate of the N10-treated group ranged from 50% to 90%. All the results shown above indicate that N10 is a multifunctional AMP. Our research broadens the source of AMPs and provides a foundation for further research into AMPs. Unfortunately, our research still has limitations here, and future studies will incorporate a broader and more diverse strain panel, with particular focus on clinical isolates carrying N10-derived MBLs across different MBL types, to more comprehensively evaluate the antimicrobial efficacy of N10.

Currently, there are four classic membrane breakage mechanisms: the barrel-stave model (41), the toroidal pore model (42), the carpet/detergent-like model, and the aggregate/nonpore model (43). In our study, the addition of the membrane depolarizing indicator DiSC3(5) demonstrated that even 2 μg/mL N10 induced early membrane dysfunction. NPN, PI, SYTO Green, TEM, and SEM analyses revealed that N10 induces membrane permeability and structural disruption, whereas ROS, H^+^, and ATP experiments revealed intracellular stress and metabolic collapse. These effects comprehensively delineate the antibacterial mechanism of N10: surface binding → membrane depolarization → permeability increase → structural disruption → intracellular stress → bacterial death. This finding is more in accordance with the carpet/detergent-like mechanism (44).

Notably, no previous studies have systematically repurposed MBL fragments as AMP templates. Therefore, our work fills a conceptual gap and introduces a novel strategy in which resistance-associated proteins can be repurposed into antimicrobial agents, potentially expanding the peptide discovery landscape. There are several limitations in our research. The half-life in plasma/body fluids, distribution within the body, and clearance pathways require further investigation. Moreover, assessing N10 resistance requires testing with additional clinical isolates and extended passage cycles to evaluate resistance pressure. The current understanding of the mechanism is primarily based on functional assays and electron microscopy observations and lacks direct evidence at the atomic or molecular level. Whether some new drug targets can be modified into AMPs remains to be determined.

## MATERIALS AND METHODS

### Bacteria information

All the bacteria were cultured at 37°C and 180 rpm, unless otherwise stated. *Streptococcus* was cultured in tryptic soy broth (TSB) supplemented with 10% fetal bovine serum (FBS) (Sijiqing, China), *Candida albicans* was cultured in Sabouraud dextrose broth (SDB), and the others were cultured in lysogeny broth (LB). The standard strains *E. coli* ATCC 25922 and *S. aureus* ATCC 25923 were stored in our laboratory. *S. aureus* 2117, *Salmonella 241107*, *Streptococcus* SC19, *E. coli* 200, *E. coli* 250321, *Klebsiella pneumoniae* WH11, and *Candida albicans* 230001 were isolated and stored in our laboratory. The media used in this study were all purchased from Sigma-Aldrich. Peptides were synthesized through GL Biochem (Shanghai, China), with the HPLC and MS files listed in Fig. S2 available at https://data.mendeley.com/datasets/9r4rcv6skv/1 (purity ≥ 95%).

### MIC

The MIC protocol was conducted as previously described (18). In brief, overnight-grown bacteria were re-cultured in Mueller-Hinton broth (MHB) to the mid-log phase. Afterward, the strains were adjusted to a final concentration of 1 × 10^6^ CFU/mL. The N10 stock solution was serially diluted twofold in MHB to prepare a range of working concentrations (0–512 μg/mL). One hundred microliters of each concentration and 100 µL of bacteria were added to each well, with the final bacteria concentration reaching 5 ×10^5^ CFU/mL. Wells containing bacteria were used as positive (growth) controls, and wells only containing MHB were used as negative (sterility) controls. The plate was then incubated at 37°C for approximately 20 h, after which the OD_600_ was determined with a microplate reader (Thermo Fisher, America).

For serum-dependent species such as *Streptococcus* spp., the MIC assay was performed using MHB supplemented with 5% (vol/vol) FBS and 0.5% yeast extract, and the plate was then incubated at 37°C for approximately 20 h, after which the OD_600_ was determined with a microplate reader. Each experiment was repeated at least three times.

### Helix wheel, amino acid composition, 3D structure, and chemical formula of peptide N10

The helix wheel was generated using HELIQUEST online software via the website https://heliquest.ipmc.cnrs.fr/cgi-bin/ComputParams.py. The basic properties of N10 were predicted via the antimicrobial peptide database (http://aps.unmc.edu/AP/), and the three-dimensional structure was predicted using PyMOL 2.3 and Avogadro 1.2.0 software. Additionally, the chemical formula of peptide N10 was determined using ChemSketch V2021.2.1.

### Cytotoxicity of the biopeptide

The cytotoxicity protocol was performed as described previously (18). Briefly, Vero, HeLa, and RAW 264.7 cells (1 × 10^5^) of human and mouse origin, respectively, were plated in 96-well cell culture plates (Corning, American) in a 37°C CO_2_ incubator until the cells covered the bottom of the plate. The cells were then washed three times with warm phosphate-buffered saline (PBS), after which DMEM supplemented with 10% CCK8 (Beyotime, China) solution and different concentrations (0, 2, 4, 8, 16, 32, 64, 128, 256, and 512 μg/mL) of peptides was added for 2 h. The OD_450_ was then detected using a spectrophotometer according to the manufacturer’s instruction. Cells supplemented with PBS were used as a negative control, and a positive control was created with medium containing 0, 2, 4, 8, 16, 32, 64, 128, 256, and 512 μg/mL peptides and melittin. Each experiment was repeated at least three times.

### Hemolytic activity of the peptides

Fresh sheep red blood cells (SRBCs) (Sbjbio, China) were centrifuged at 4°C for 10 min at 800 rpm. The SRBCs were then washed three times with PBS until the supernatant was clear. The SRBCs were then carefully resuspended in PBS at a concentration of 10%. Blood cells were cocultured with 0, 2, 4, 8, 16, 32, 64, 128, 256, and 512 μg/mL of peptides at 37°C for 2 h. The OD_450_ of this supernatant was then determined as *A_x_*, where *A*_positive_:


$$\text{Hemolytic rate }\%=\frac{A_{x}-A_{0}}{A_{positive}-A_{0}}\times 100\%$$

Each experiment was repeated at least three times.

### Assessment of bacterial resistance

*E. coli* ATCC 25922 and *E. coli* 250321 were cultured as described previously, and the amount of bacteria was adjusted and coincubated with 0.5× MIC concentration of meropenem or N10. After being incubated for 24 h, the MICs of the surviving bacteria were determined. Afterward, the same steps were performed 30 times in 30 days, and the MIC values were used to plot the resistance curve. Each experiment was repeated at least three times.

### Survival rate

Bacteria grown overnight were added to fresh LB at a ratio of 1:100 and washed three times with PBS after the bacteria had grown to the mid-log phase at 37°C (21). The bacteria were then adjusted to 1 × 10^8^ with PBS, and 0.25×, 0.5×, 1×, and 2× MIC N10 were added to the bacteria at 37°C. After 1 h, 10× serial dilutions were used to count the CFUs, which were labeled B1. The unamended group was the negative control and was labeled B0. The survival rates of the bacteria were determined according to the following formula. Each experiment was repeated at least three times.


$$\text{Survival rate }\%=\frac{B_{1}}{B_{0}}\times 100\%$$

### Time‒kill curve

After the bacteria were washed with PBS and adjusted to approximately 1 × 10^8^, the 1× MIC of N10 was added, and the mixture was cultured at 37°C. A total of 100 μL of bacteria was subjected to 10× serial dilution every 1 h. The CFUs were counted after 12 h, and each assay was performed at least three times.

### Growth curve

Bacteria grown overnight were transferred to fresh medium at a ratio of 1:100, and 1× MIC N10 was added to the medium. A total of 200 μL of the mixture was added to a 100-well growth curve plate and placed in a fully automated growth curve detector (Finland) (18). The OD_600_ was continuously monitored for 12 h, and the data were recorded every hour. The assay was repeated at least three times.

### Structured illumination optical microscopy

For the biofilm integrity assay, the steps were the same as previously described (19). The samples were stained according to the instructions for FilmTracer LIVE⁄DEAD Biofilm Viability Kit (Thermo Fisher, America). The images were then captured using a fluorescence microscope (Nihonika, Japan).

For the bacterial integrity assay, bacteria were grown to the mid-log phase and then were washed three times with PBS. Bacteria and 0.5× MIC of N10 were cocultured at 37°C for 30 min and then washed three times with PBS. The samples were stained with FITC-N10 and DAPI for approximately 15 min each. After the samples were washed with PBS three times, images of the bacteria were captured via fluorescence microscopy (Nihonika, Japan).

### Scanning electron microscopy

Bacteria cultured overnight were transferred to fresh medium when they reached the mid-log phase and were subsequently washed three times with PBS. Then, 500 µL of PBS was used to resuspend the bacteria, after which 0.5× MIC of N10 was added, and the bacteria were cultured at 37°C for 1 h. The bacteria were subsequently washed three times with PBS and centrifuged at 4,000 rpm for 5 min. The cells were fixed with 2.5% glutaraldehyde at room temperature for 2 h and then incubated at 4°C overnight. The cells were dehydrated, sprayed with gold, and visualized by scanning electron microscopy (HITACHI Regulus 8100, Japan).

### Biofilm formation

The bacteria, which had been cultured overnight, were then grown at the mid-log phase in fresh medium and adjusted to an OD_600_ = 1.0. Two hundred microliters of bacteria were added to a 96-well plate. Moreover, the bacteria were mixed with 1, 5, or 10 μg/mL N10. The blank medium was used as a negative control. After 36 h of incubation at 30°C, the plates were gently washed three times with PBS. Then, 100 µL of methanol was added, and the samples were fixed at room temperature for 15 min. Afterward, the biofilms were stained with 1% crystal violet for 10 min at room temperature. The residual crystal violet was then removed by washing with 200 µL ddH_2_O, after which the plate was dried at room temperature. Finally, 100 µL of 33% ethanol was added to dissolve the crystal violet, and the OD_590_ was detected with a spectrophotometer (FLUOSTAR OMEG, USA). Each assay was performed at least three times.

### ROS content determination

*E. coli* ATCC 25922 was cultured overnight, transferred, and grown to mid-log phase. After being washed and resuspended in PBS three times, DCFH-DA (Thermo Fisher, USA) was added to a final concentration of 10 μmol/L, and the mixture was incubated at 37°C for 30 min. After being washed with PBS, the bacteria were treated with different concentrations of N10 and incubated for 1 h. The mixture was aspirated into a 96-well black ELISA plate (Corning, USA) for fluorescence signal acquisition. Each assay was performed at least three times.

### ATP leakage

Bacterial culture was performed as described above, with the addition of 5 or 10 μg/mL N10 to the bacteria. After 1 h of incubation at 37°C, the bacteria were centrifuged at 5,000 rpm for 5 min, and the supernatant and pellet were collected. Cell lysis buffer was then used to lyse the pellet, firefly luciferase was added to both solutions according to the product instructions (Byotime, China), and these groups were labeled as extracellular or total. After the steps described above, the fluorescence of the sample was detected using a multimode reader (SPARK10M, America). Afterward, the standard solution was prepared, and the content of total or extracellular ATP content was calculated. Each assay was performed at least three times.

### Membrane potential assay

The membrane potential assay was monitored by using NPN (Merck, America), PI (Merck, America), SYTOX Green (Thermo Fisher, America), and DiSC3(5) (MCE, America), as described previously with minor modifications (45). Mid-log-phase cells were washed and adjusted to an OD_600_ of 0.5 in HEPES buffer supplemented with 100 mM KCl, followed by incubation with 0, 2, 4, 8, and 16 μg/mL of N10. The cells were then cultured with NPN, PI, or DiSC3(5) at 37°C in the dark for 1 h. Afterward, the cells were vortexed, and 200 μL of the mixture was transferred to black 96-well microplates. Changes in fluorescence were recorded using a fluorescence microplate reader with excitation (Ex): 350 nm, emission (Em): 420 nm (NPN), Ex: 535 nm, Em: 617 nm (PI), Ex: 488 nm, Em: 523 nm (SYTOX Green) or Ex: 622 nm, Em: 670 nm. Each assay was performed at least three times.

### Cytokine measurement assay

The cell culture steps were the same as those described previously (18). HeLa cells were grown in 24-well plates, and *E. coli* 250321 was used to infect the cells at an MOI of 10 for 1 h. The supernatant and cells were collected separately, after which the IL-1β (Beyotime, China), IL-6 (Beyotime, China), and TNF-α (Beyotime, China) levels were detected.

The collected cells were lysed, and RNA was extracted with a TransGen (China) EasyPure RNA Kit manufacturer’s instructions. The fresh RNA was then reverse-transcribed using EasyScript One-Step RT-PCR SuperMix manuscript instructions. The templates were then used to perform quantitative PCR (qPCR) with PerfectStart Universal Green qPCR SuperMix according to the instructions. The PCR process was completed on a Bio-Rad CFX96 machine (USA). β-actin was used as a control, and the primers used in this study are listed in Table S2 available at https://data.mendeley.com/datasets/9r4rcv6skv/1. Each assay was performed at least three times.

### Animal experiments

All the animal experiments were performed through standard procedure, and the experimental protocols were approved by Heze University.

For the survival rate experiment, 40 BALB/c female mice weighing 20 ± 2 g were grouped into *E.coli* 250321 infection, 0.5×, 1×, and 2× MIC N10 treatment groups. Then, 1 × 10^7^ fresh *E.coli* 250321 cells were vaccinated through intraperitoneal injection. The treatment process was performed described for the model procedure, and mouse deaths were recorded every day.

For the tissue load experiment, 15 BALB/c female mice weighing 20 ± 2 g were grouped into 3 groups (5 in each group). A total of 1 × 10^7^ fresh *E. coli* 250321 cells were vaccinated in the same way. Different concentrations of N10 were then injected after infection for 2 h and 12 h. Blood was taken through orbital blood sampling, and the mice were anesthetized until death. The heart was perfused with PBS until the blood was clean; after which, the tissue was cut and ground. Then, 10× series dilutions were performed, and the cells were cultured overnight at 37°C. The CFUs were then counted, and the tissue load was subsequently calculated.

### Statistical analysis

The data mentioned above were analyzed using GraphPad Prism 7.0. To assess the means between two distinct groups, Student’s *t*-test was employed. ANOVA was applied to compare the means across multiple groups. *, *P*<0.05; **, *P*<0.01; ***, *P*<0.001; ns indicates nonsignificance.

## Data Availability

The data supporting this study have been deposited in Mendeley Data and are publicly accessible via DOI: 10.17632/9r4rcv6skv.1.
